# Artificial nighttime lighting impacts* Plasmodium falciparum* mature stage V gametocytes infectivity in *Anopheles stephensi*

**DOI:** 10.1186/s12936-024-04866-6

**Published:** 2024-02-08

**Authors:** Jose Luis Llergo, Helena Garuti, Celia Lopez, Julia Sanchez, David Calvo

**Affiliations:** 1 Global Health Medicines R&D, GlaxoSmithKline, C/Severo Ochoa 2, Tres Cantos, 28760 Madrid, Spain; 2 In Vivo Science and Delivery (IVSD), GlaxoSmithKline, C/Severo Ochoa 2, Tres Cantos, 28760 Madrid, Spain

**Keywords:** *Anopheles stephensi*, *Plasmodium falciparum*, Artificial light at night, SMFA, Prevalence, Malaria

## Abstract

**Background:**

Malaria is one of the most important vector-borne diseases of humans with an estimated 241 million cases worldwide in 2020. As an urban and periurban mosquito species, *Anopheles stephensi* is exposed to artificial human stimuli like light that can alter many aspects of mosquito behaviour, physiology and metabolism. Therefore, fluctuations in the light environment may influence the host, parasite and/or mosquito biology and hence modulate risk for disease transmission. In this study, the effect of artifitial light at night on mosquito infectivity by *Plasmodium falciparum* during the first hours of blood digestion was tested.

**Methods:**

A total of three independent standard membrane feeding assays were performed to artificially fed septic and aseptic mosquitoes with *P. falciparum* infected blood. After blood feeding, females were transferred to incubators with different photoperiod cycles, so digestion occurred under day artificial light or dark. At 7 and 16 days post blood feeding, mosquitoes were dissected for midguts and salivary glands, respectively. Percentage of mosquitoes fed, percentage of prevalence and *P. falciparum* oocyst intensity between septic and aseptic mosquitoes in the two different photoperiod regimes, were compared using a Kruskal-Wallis test followed by a Dunn´s multiple comparison test .

**Results:**

The exposition of mosquitoes to light after they took an infected blood meal has a negative effect on the successful progression of *P. falciparum* in the mosquito midgut. Antibiotic treatment significantly incremented the number of oocysts per midgut. Photophase significantly reduced the median oocyst intensity in both septic and aseptic mosquitoes. The percentage of oocyst reduction, understood as the percentage of reduction in the mean oocyst intensity of the parasite in the mosquito midgut between photophase and scotophase, was 51% in the case of aseptic mosquitoes and 80% for septic mosquitoes, both in the photophase condition.

**Conclusion:**

Although there are still many gaps in the understanding of parasite-mosquito interactions, these results support the idea that light can, not only, influence mosquito biting behaviour but also parasite success in the mosquito midgut. Hence, light can be considered an interesting additional mosquito-control strategy to reduce mosquito-borne diseases.

## Background

Malaria is one of the most important vector-borne diseases of humans with an estimated 241 million cases worldwide in 2020, being Africa the region that recorded the 95% of the malaria cases and the 96% of deaths worldwide [1]. A huge effort was made to control this devastating disease worldwide over the past decades, but the emergence of drug-resistant parasites as well as vector insecticide-resistant, make it imperative to develop new approaches to target the disease. Developing medicines aiming for the transmission stages of the malaria parasite in the mosquito is one of the main strategies for global malaria eradication [2].

From the several hundreds of mosquito species described worldwide, approximately 40 species of the genus *Anopheles* are known to be competent for malaria parasites [3]. Among them, *Anopheles stephensi* is a major malaria vector, for both *Plasmodium falciparum* and *Plasmodium vivax,* with a geographical distribution extending from the Middle East through the Indian subcontinent to China [4]. Since 2012, this species has been detected in Africa, where it has become a major potential threat to malaria control and elimination due to its rapid propagation in urban and suburban areas that have produced an increase in the number of malaria cases [5].

All organisms have evolved internal timekeepers (circadian rhythms) that allow them to predict daily and seasonal changes in light, temperature, chemicals and/or social cues [6]. Light and temperature have been considered to be two of the most important abiotic factors affecting endogenous rhythms. Artificial Light at Night (ALAN), which is the light from anthropogenic sources, has an important effect on insect biology, from genes to organismal physiology and behaviour [7]. In the case of *An. stephensi* mosquitoes with an urban and periurban distribution, the exposition of artificial human stimuli, like artificial light, can affect many aspects of anophelines biology like oviposition, sugar and blood feeding, biting patterns and locomotor activity [6]. Circadian rhythms not only affect the vector but also affect parasite biology. First of all, the expression of physiological and immune host genes that are affected by the circadian rhythms may potentially impact the development of the parasite. Further, many vector-borne pathogens may have evolved to match the daily fluctuations in vector abundance to assure their transmissibility, i.e., potential inducers of gametocyte maturation may be under circadian control [8]. Therefore, fluctuations in the light environment may influence the host, parasite or mosquito biology and hence modulate risk for disease transmission [9].

For transmission to succeed, *Plasmodium* gametocytes must be taken up by females *Anopheles* during its blood meal. In the mosquito midgut, lumen fertilization occurs within the first hour post-feeding. From 8 to 24 h later, the zygote differentiates into a motile ookinete, moves out of the blood bolus and migrates across the peritrophic matrix until the basal side of the epithelial cells where the oocyst will develop. Since *Anopheles* are primarily night biters, all the fertilization, ookinete migration and oocyst formation is happening in a dark environment [10]. However, in urban and suburban areas, the nighttime environment has been altered by ALAN, which may be changing aspects of mosquito biology as well as influencing the parasite sexual development in the mosquito midgut, thus having an effect on the vectorial capacity of the mosquito. In this work, the effect of ALAN on the infectivity of mosquitoes by *P. falciparum* during the first hours of blood digestion was tested. For this purpose, mosquitoes were artificially fed through Standard Membrane Feeding Assay (SMFA) with *P. falciparum* infected blood and transferred afterwards to incubators with different photoperiod cycles. In that way, mosquito digestion occurred under day artificial light or under dark which allowed to determine the effect of ALAN in the mosquito infection through the determination of oocyst and sporozoite load in the mosquito midgut and salivary glands, respectively.

## Methods

### Gametocyte and mosquito production

Gametocyte cultures were generated from *P. falciparum* NF54 strain from BIE Resources, USA. Gametocyte culture protocol was adapted from that described by Ifediba and Vanderberg [11]. Briefly, gametocyte induction was initiated at 0.5% parasitaemia (> 70% rings) and 4% haematocrit in RMPI medium (Sigma-Aldrich^®^, R5886) with 5% human serum A^+^ (TCS Scientific, UK) and 5% Albumax (Gibco # 11021–0379). Cultures were maintained with daily media change and without addition of fresh RBCs. Percentages of mature gametocytes were assessed by microscopic counts (Leica, DM2000) of Giemsa-stained thin blood smears. Cultures were checked at day 14 for stage V gametocytaemia, male and female gametocyte ratio and number of male exflagellations. At day 16, cultures with a stage V gametocytaemia between 1 and 3%, male/female ratio of not less than 1:2 and exflagellations of not less than 20 centres/field were used to feed mosquitoes.

The *An. stephensi* (sda-500 strain) [12] colony was established in 2014 from eggs kindly supplied by Michael Delves and Mark Tunnicliffe at Imperial College, London. Eggs, larvae and adults were maintained in climate-controlled chambers (Panasonics MLR 352-H) at a temperature of 26.5 ± 1 °C, 14L: 10D photoperiod and a relative humidity of 75 ± 5%. Between 2 and 3 days before the experiments were performed, mosquitoes were transferred to small cups in a density of 40 females/cup. In order to remove the mosquito bacterial flora in the midgut, part of the mosquitoes used for experiments were fed daily with a mixture of antibiotics (50 mg/mL gentamicin solution [Sigma-Aldrich^®^, G1397] and penicillin–streptomycin solution [Sigma-Aldrich^®^, P4333]) in a 10% sterile sucrose solution. Antibiotic treatment was maintained from day zero until 24 h before the feeding (a maximum of 4 consecutive days). Before blood feeding, the antibiotic-sucrose solution was removed, and mosquitoes were starved for 24 h. A control cage was maintained in the same conditions but without antibiotics. Control mosquitoes without antibiotic treatment were maintained in the same conditions, transferred to small cups and put on starvation 24 h before blood feeding. After antibiotic treatment mosquitoes became aseptic and are referred as aseptic mosquitoes, while untreated mosquitoes are referred as septic.

### Standard membrane feeding assay (SMFA)

A total of three independent SMFAs were performed. For each SMFA, cups with 40 *An. stephensi* female mosquitoes treated and un-treated with antibiotics, between 4 and 6 days old, were fed with a *P. falciparum* gametocytes-enriched blood meal for a duration of 30–40 min via Parafilm^®^ membrane attached to glass feeders (Fisher Scientific, #12831283) connected to a 37 ºC circulating water bath. Since the biting activity of *An. stephensi* is higher during the first quarter of the night, all the SMFA were performed in the dark as a way to increase proportion of mosquitoes fed in the experiments. After blood feeding, septic as well as aseptic mosquitoes were equally divided and transferred to different Panasonic incubators that were in different photoperiod phases: photophase (light phase) and scotophase (dark phase). In the incubator with the photophase condition, the cycles started with 13.5 h of full light at 8:00 am and 9.5 h darkness starting at 9:30 p.m., separated by 0.5 h dawn and dusk transitions, respectively. Conversely, the incubator in the scotophase, the cycles started with 9.5 h of darkness at 8:00 am and 13.5 h full light starting at 6:30 p.m., separated by 0.5 h dawn and dusk transitions, respectively. All SMFAs performed took place between 11:00 a.m. and 12:30 p.m. At 24 h after mosquito feeding, unfed ones were counted and removed from each cup which allowed us to calculate the percentages of mosquitoes fed in each experiment.

At 7 days post blood feeding, mosquitoes were dissected for midguts using a stereomicroscope and stained in 0.2% mercurochrome solution for 10 min. Total number of oocysts in individual midguts were counted using a light microscope (Leica, DM2000) using a 10X Objective (100X magnification). Both, infection prevalence (percentage of mosquitoes with one or more oocyst) and mean oocyst intensity of infection are defined for all the conditions tested. A subsample of the mosquitoes for each condition was maintained until day 16 post blood feeding when sporozoite dissections were performed.

The % of oocyst reduction (OR) and the % of block in transmission (BiT) was calculated as follows:

$$OR\%=100-\left(\frac{{OM}_{L}*100}{{OM}_{D}}\right)$$, where.

OR% = Oocyst Reduction Percentage.

OM_L_ = Oocyst Mean Intensity in mosquitoes in photophase.

OM_D_ = Oocyst Mean Intensity in mosquitoes in scotophase.

$$BiT\%=100-\left(\frac{{P}_{L}*100}{{P}_{D}}\right)$$, where.

BiT% = Block in Transmission Percentage.

P_L_ = Infection Prevalence in mosquitoes in photophase (% of infected mosquitoes).

P_D_ = Infection Prevalence in mosquitoes in scotophase (% of infected mosquitoes).

### Sporozoite dissection

Between day 16 to day 20 post blood feeding, mosquitoes were anesthetized in a CO_2_ chamber for 2 min and kept on ice for dissection. Later, salivary glands were dissected under a stereomicroscope using cold RPMI medium (Sigma-Aldrich^®^, R5886). All salivary glands from the same condition tested were pooled together in a cold 1.5 ml Eppendorf tube with 80 µl of RPMI medium. Salivary glands were dissected during a maximum time of 1.5 h. After a short spin (1000 rpm for less than 1 min), the samples were crushed with a sterile pestle. 10 µl of a sporozoites suspension was placed into a Neubauer chamber haemocytometer (INCYTO DHC N015, Fisher Scientific). A phase contrast microscope, set to 40 × objective (400X magnification), was used to enumerate the sporozoites. The number of sporozoites was counted in the four quadrants of the chamber and, the total number of sporozoites was calculated, using the formula:

$$\frac{(four \,quadrants)}{4}=\#of \,sporozoites \times {10}^{4}/ml$$

### Statistical analysis

Data were analysed using GraphPad Prism 6 (GraphPad Software, La Jolla, California, USA). Percentage of mosquito fed, percentage of prevalence and *P. falciparum* oocyst intensity between septic and aseptic mosquitoes in the two different photoperiod regimes were compared using a Kruskal–Wallis test followed by a Dunn´s multiple comparison test. Comparisons of the number of sporozoites in aseptic mosquitoes between the scotophase and photophase were performed using Mann–Whitney U test.

## Results

*Plasmodium falciparum *in vitro gametocytes production parameters are shown in Table 1. Asexual stage parasites at the day of induction were in a range of 4–6%, with a ring percentage at induction close to 100% in all the assays performed. At the day of the SMFA, stage V gametocytaemia was higher than 1% with a male:female ratio of more than 2 females for male and, with a number of exflagellation centres/field higher than 47.

**Table 1:** *P. falciparum *in vitro culture parameters

	In vitro culture paramaters		
	Exp 1	Exp 2	Exp 3
Parasitemia at induction (%)	5	4	6
Ring percentage at induction (%)	100	96	92
Age on day of SMFA (days)	15	16	15
Gametocyte Stage V (%)	1.10	1.73	1.10
Male: Female	1:2.3	1:2.2	1:2.5
RBCs 10 6 /ml	272	270	308
Exflagellation/field in the SMFA^(1)^	47	88	52

The table depicts the different parameters recorded during the in vitro production of *Plasmodium falciparum* stage V gametocytes that were used to infect mosquitoes through the Standard Membrane Feeding Assay (SMFA) for three independent experiments

^(1)^Count by eye under microscope when SMFA are performed

**Fig. 1:**
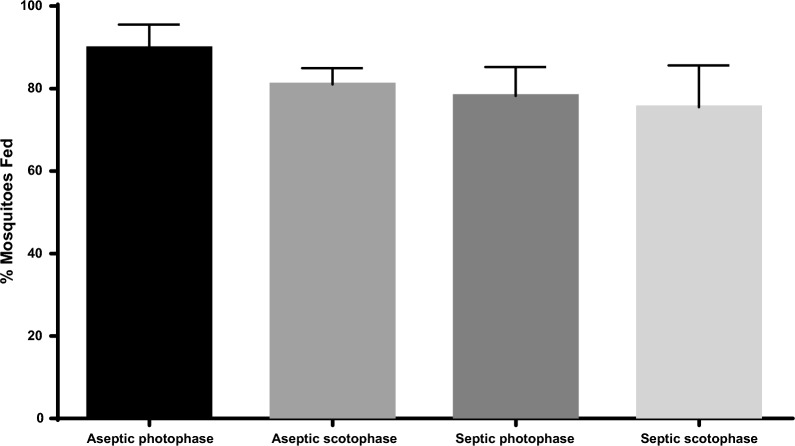
Total percentage of mosquitoes fed on infected blood with *P. falciparum* mature stage V gametocytes through the SMFA. Graphic shows the total percentage of mosquitoes fed in three independent experiments. The bars show the mean ± SD. Aseptic = Mosquitoes treated with antibiotics since emergence until 24 h before blood feeding; Septic = Mosquitoes non-treated with antibiotics; Photophase = After blood feeding, mosquitoes were transferred into an incubator in light phase; Scotophase = After blood feeding, mosquitoes were transferred into an incubator in dark phase. Kruskal–Wallis test followed by a Dunn Test for Multiple Comparisons was used to compare the different experimental groups (groups that were statistically significant are represented with an asterisk: * p < 0.1, ** p < 0.01, *** p < 0.001, **** p < 0.0001). Barless conditions were also subjected to analysis but were not significantly different

All the SMFAs were performed in the dark to avoid any alteration of *An. stephensi* biting behaviour. The percentage of mosquito feeding was higher than 75%, with no significant differences among conditions (Kruskal–Wallis test: p ≥ 0.05, H = 7.8, DF = 3). Antibiotic treatment, and as a consequence the removal of the microbiota, did not affect mosquito feeding, with both aseptic as well as septic mosquitoes showing similar percentages of feeding (Fig. 1).

**Fig. 2:**
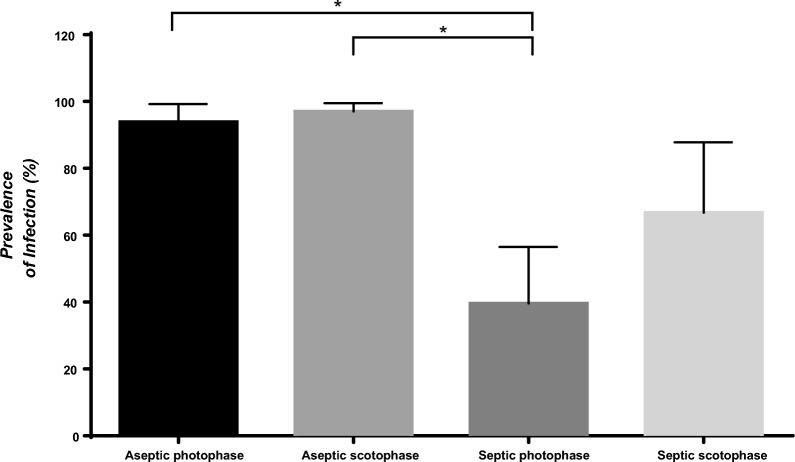
Prevalence of infection of mosquitoes fed on infected blood with *P. falciparum* mature stage V gametocytes through the SMFA. Graphic shows the prevalence of infection of mosquitoes fed in three independent experiments. The bars show the mean ± SD. Aseptic = Mosquitoes treated with antibiotics since emergence until 24 h before blood feeding; Septic = Mosquitoes non-treated with antibiotics; Photophase = After blood feeding, mosquitoes were transferred into an incubator in light phase; Scotophase = After blood feeding, mosquitoes were transferred into an incubator in dark phase. Kruskal–Wallis test followed by a Dunn Test for Multiple Comparisons was used to compare the different experimental groups (groups that were statistically significant are represented with an asterisk: * p < 0.1, ** p < 0.01, *** p < 0.001, **** p < 0.0001). Barless conditions were also subjected to analysis but were not significantly different

Aseptic mosquitoes showed higher prevalence of infection compared with septic mosquitoes independently of the phase (scotophase or photophase) they were maintained after a blood meal (Kruskal–Wallis test: p < 0.01, H = 12.5, DF = 3) (Fig. 2). No significant differences among scotophase/photophase conditions were detected in prevalence for aseptic mosquitoes. In the case of septic mosquitoes, although without significant differences, septic mosquitoes in the photophase showed lower prevalence of infection.

**Fig. 3:**
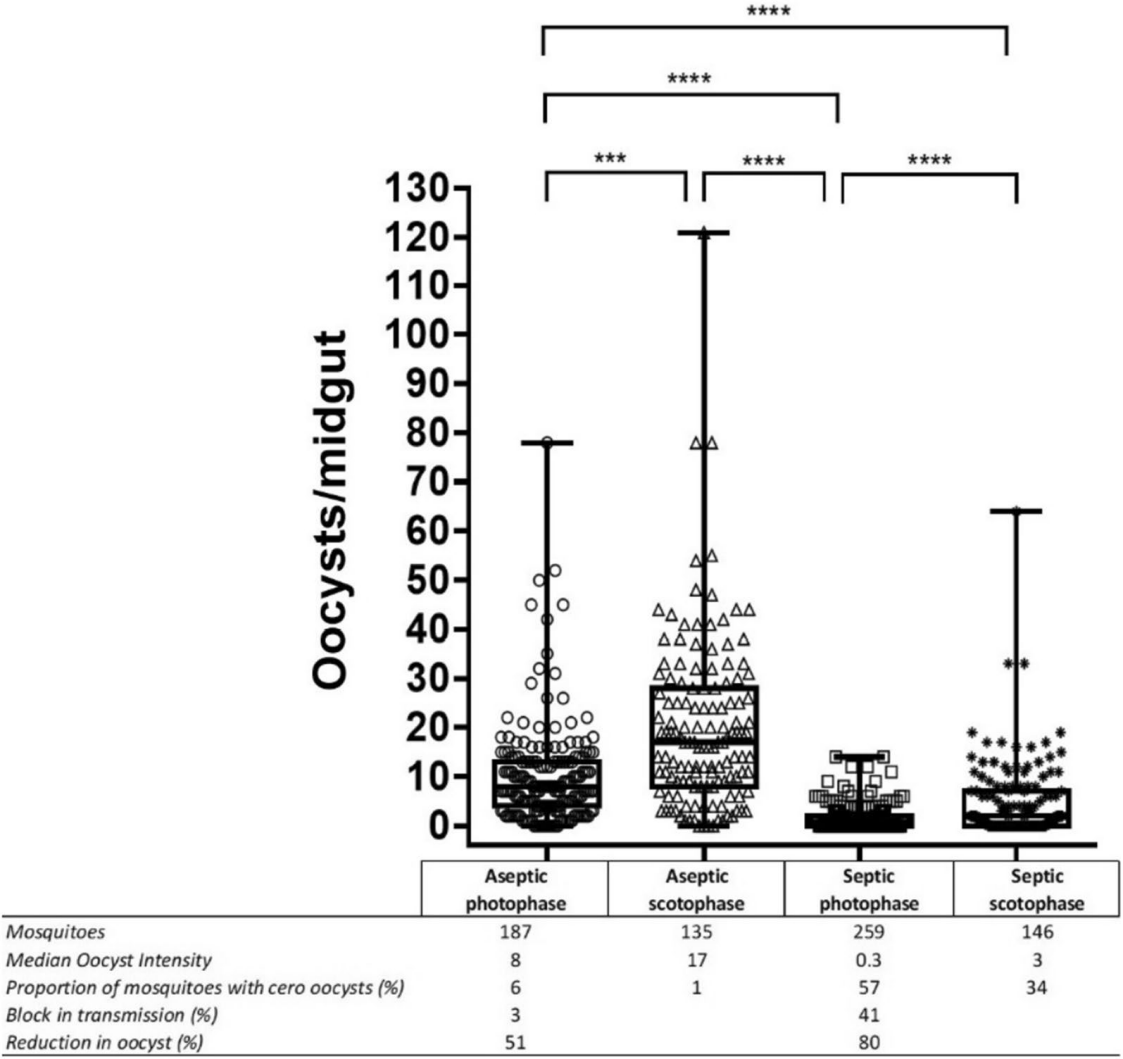
*P. falciparum* oocyst intensity of mature GC in the SMFA. Each dot represents the total number of oocysts in a single mosquito midgut. The black box shows the interquartile range, the line in the box depicts the median oocyst intensity of infection, and the top and bottom whiskers show the highest and the lowest value respectively. Figure represents results from three independent experiments. Kruskal–Wallis test followed but a Dunn´s Multiple Comparison Test was used to compare the statistical significance between the different experimental groups (groups which showed a statistically significant difference are represented with asterisks: p < 0.1, p < 0.01, p < 0.001, p < 0.0001 is represented by *, **, ***, **** respectively). Barless conditions were also subjected to analysis but were not significantly different. The table below the graph depicts the total number of full-fed mosquitoes dissected per condition and the % mean oocyst intensity, reduction in mean oocyst intensity and the % of block in transmission. Graphics represent results from three independent experiments

At day 7 post blood feeding, mosquitoes from the different conditions were dissected for midguts to determine the oocyst load. Antibiotic treatment (aseptic mosquitoes) incremented significantly the number of oocysts per midgut (Fig. 3) (Kruskal–Wallis test: p < 0.0001, H = 330.2, DF = 3). Photophase significantly reduced the median oocyst intensity in septic as well as in aseptic mosquitoes. In the case of aseptic mosquitoes, the median oocyst intensity was 2.12 times lower under photophase than under the scotophase. Similar pattern was detected for septic mosquitoes, where tenfold reduction was detected in the median oocyst intensity between photophase and scotophase (Fig. 3).

The percentage of oocyst reduction, understood as the percentage of reduction in the mean oocyst intensity of the parasite in the mosquito midgut between photophase and scotophase, was 51% in the case of aseptic mosquitoes and 80% in the case of septic mosquitoes in the photophase condition (Fig. 3). The percentage of block in transmission, parameter that is based on the percentage of prevalence, a 3% of block in transmission was recorded for aseptic mosquitoes in the photophase compared to the aseptic mosquitoes in the scotophase. In the case of septic mosquitoes, the % of block in transmission was 41% (Fig. 3). This effect on parasite reduction in the midgut was also reflected in the proportion of mosquitoes in each condition with zero oocysts in their midguts after receiving an infected blood meal. For aseptic mosquitoes this proportion increased from 1% for the scotophase to 6% for the photophase condition (Fig. 3). For septic mosquitoes, similar tendency was detected, with 57% of the septic mosquitoes in the photophase with zero oocysts compared with a 34% for the septic mosquitoes in the photophase condition. These results reflected a clear effect of light in the reduction of the proportion of mosquitoes infected with *P. falciparum*.

Sporozoites in the salivary glands were determined only for aseptic mosquitoes. No salivary glands were dissected for septic mosquitoes due to the low infection recorded. No significant differences were detected in the total sporozoites load obtained between aseptic mosquitoes in the photophase and the scotophase condition (Mann–Whitney U test, p = 0.86). The total sporozoite load per aseptic mosquito in the photophase condition was 4208 ± 850 (mean ± SE) from a total of 187 mosquitoes dissected. For aseptic on the scotophase, the total sporozoite load per mosquito was 5320 ± 1838 (mean ± SE) from a total of 137 mosquitoes dissected.

## Discussion

Human development has altered large areas worldwide that impacted animal biology and behaviour. One of those alterations is the use of artificial light sources which have transformed the nighttime environment [13]. In the case of *An. stephensi,* which adults are predominantly anthropophilic, endophilic and endophagic, with a predominantly crepuscular and nocturnal biting activity, the impact of scotophase and photophase during the critical first 24 h of blood digestion in the mosquito midgut after they were fed with plasmodium infected blood was experimentally adressed. Results showed that the alteration of nighttime patterns by the use of artificial light had an effect in the vector-parasite interactions that translated into a reduction in vector competence.

A significantly different vector competence for *P. falciparum* between septic and aseptic mosquitoes was recorded. It has been widely described that when female anopheline mosquitoes ingest gametocytes from a *Plasmodium* infected blood meal, the parasite has to overcome a strong population bottleneck due to low fertilization efficiency and the pressure of the vector´s innate defense mechanisms. During the first 18–24 h after the blood meal- what is called the “early phase”- the pressure will be exerted mainly against the ookinete stage [14–17]. During this early phase, midgut bacteria have an indirect role in parasite interference through the induction of antimicrobial peptides (AMPs) and other immune specific genes. For example, it has been proved in *Aedes aegypti* that the absence of gut bacteria reduces AMPs and ROS (reactive oxygen species) expression promoting mosquito infectivity by *Plasmodium* [18]. At the same time, the blood ingestion itself also produced an up- or down-regulate genes involving immunity related gens or oxidative stress and redox genes in *Anopheles gambiae* [19]. The invasion of gut cells by the ookinete, called “late phase”, will induce nitric oxide synthase (NOS) expression, resulting in high levels of nitric oxide (NO). This will imply the production of reactive oxygen species as well as the activation of the mosquito complement-like system [20], targeting the early phases of oocyst formation and reducing significantly the number of mature oocysts [21, 22]. As described previously in other mosquito species [23], the elimination of midgut bacteria in *An. stephensi*, and as a consequence the lack of peritrophic membrane formation [24], by antibiotic treatment promoted *Plasmodium* infectivity in the mosquito by an increment of 20% in prevalence as well as four times increment in the median oocyst intensity (see Fig. 3 in the results section).

When the presence of light was added to the equation, septic and aseptic mosquitoes maintained in a photophase during the first hours of blood digestion showed significantly different vector competence to *P. falciparum* than the ones maintained in a scotophase. This effect of light was more pronounced in septic than aseptic mosquitoes which pointed out to a strong effect of the microbiota against the parasite, which is enhanced during the photophase. Insects have evolved to respond to the daily light:dark cycles that produced rhythms in ambient light, temperature, humidity, UV radiation and resource availability [7, 9]. Light acts as a prominent environmental cue to regulate most of the mosquito’s biological processes from gene expression to physiology and behaviour, like for example biting, molecular clock genes, sugar feeding, metabolism or immunity among others [9, 19, 25, 26]. In the other hand, it has been also described that microbiota present in the mosquitoes as well as the development of *Plasmodium* are subjected to light:dark rhythms [27]. Bacteria community in the mosquito shifts during life stage transitions and community structure is associated with dietary regimes as well as mosquito species [28, 29]. During sugar feeding, the gut receives a carbohydrate-rich but protein limited content. The opposite scenario is found after blood feeding. This different nutritional environment will shift the compositional microbiome in the gut [28, 30]. Therefore, the catabolism of blood meal will reshape the bacteria community with the expansion of those bacteria capable of coping with the oxidative stresses on the bolus. Considering that gut microbiome has also a link with the immune system in the mosquitoes and that light can regulate the expression of different immunity, oxidative stress, redox and metabolism genes [15, 31], the combined effect of both factors may produce a significant reduction in *Plasmodium* infectivity in the mosquito. At the same time, gut commensal bacteria should also have a differential expression in function on the period of the day which will shape the bacteria population in the midgut accordingly to the period of the day and the mosquito behaviour.

The expression of immune-related genes that encodes regulator of immune deficiency (IMD) pathway and several immune effectors like defensin 1, cecropin or nitric oxide synthase (NOS) have been shown to present rhythmic expression [25], being regulating by blood feeding as well as environmental cues like light that can alter organismal physiology and behaviour [26]. For example, Das and Dimopoulos [19] recorded that in *An. gambiae*, blood digestion as well as light up-regulated the aminopeptidase genes, which in previous studies have been involved in blocking the development of *Plasmodium* ookinetes. According to the results obtained, similar patterns of genes regulation have to be happening and affecting parasite infectivity producing the significant reduction of 80% in oocyst intensity in septic mosquitoes in the photophase compared with septic mosquitoes maintained in the scotophase. This effect was also recorded in aseptic mosquitoes, but the reduction in oocyst was only 51%, pointing out that in this case the effect will be only due to the regulation exerted by blood digestion to gene expression, since bacteria were removed from those mosquitoes. Therefore, the exposition of mosquitoes to light after they took an infected blood meal will have a negative effect on the successful progression of *P. falciparum* in the mosquito midgut. In the case of sporozite production, although no sporozoite dissection were performed for septic mosquitoes due to the low infectivity, the effect of light did not seem to have a detectable effect on sporozoite production, since no significant differences were detected in the number of sporozoites produced between aseptic mosquitoes in the photophase or socotophase.

## Conclusion

Light affected parasite progression in the mosquito during one of the main bottlenecks, the ookinete stage. This effect was increased when microbiota was present in the mosquitoes by 30% increment in oocyst reduction. The effect of light is remarkably important in the case of *An. stephensi* since its distribution in urban and peri-urban areas exposed them to artificial light. Although still there are a lot of gaps in the understanding of parasite-mosquito interactions, the results support the idea that light can not only influence mosquito biting behaviour but also parasite success in the mosquito midgut. Hence, light need to be consider an interesting additional mosquito-control strategy to reduce mosquito-borne diseases.

## Data Availability

The datasets used and/or analysed during the current study are available from the corresponding author on reasonable request.
